# Collaborative research in surgery: a rising tide lifts all boats

**DOI:** 10.1093/bjs/znac099

**Published:** 2022-04-14

**Authors:** Elizabeth Li, Aneel Bhangu

**Affiliations:** NIHR Global Health Research Unit on Global Surgery, University of Birmingham, Birmingham, UK; NIHR Global Health Research Unit on Global Surgery, University of Birmingham, Birmingham, UK

Collaborative research is not about authorship. It is about delivering rapid benefit to patients and engagement of wider communities. Single-centre publications of highly selective, niche populations are common, but inefficient and weak. Research that unnecessarily excludes women, the elderly, or ethnic groups further reduces the validity of any findings and cannot be applied to other settings. The balance of power skewed towards a small number of top-line authors removes benefits from patients. Finally, research using data that are many years old is increasingly lacking relevance. Collaborative research solves these problems, and fundamentally prioritizes delivering tangible outputs on behalf of patients. Any benefits to surgeons are secondary.

Collaborative research studies are multicentre and often multicountry^1–4^. This means a diverse range of patients is included, typically from different resource settings and socioeconomic statuses. This makes the findings representative of wider populations, meaning that the results are generalizable and relevant to more patients. The scale of collaborative studies can efficiently generate high-quality prospective data while maintaining a low burden of commitment for individual collaborators. The subsequent large data sets reduce outlying results, provide the opportunity to answer multiple research questions, and are relevant to numerous subgroups of patients. In short, collaborative research is a highly efficient method of generating research data using streamlined and available resources.

As a community, collaborative researchers are also learning to publish more rapidly. Working with journal editors, the release of data is fast approaching real-time findings. This has been shown to be invaluable in rapidly evolving situations, the most recent and prominent example being the COVID-19 pandemic^1,2,5^.

Collaborative research is not restricted by study type or structure, and is both randomized and non-randomized. The FALCON^2^, ROSSINI^6^, FOxTROT^7^, and STAR-TREC^8^ trials are examples of collaborations that are producing practice-changing research. FALCON completed recruitment of 5800 patients in 24 months and published within 3 months of closing. It is relevant to patients all over the world. The COVIDSurg Collaborative is an example of non-randomized collaborative study^1,3,5^. In the wake of the COVID-19 pandemic, these studies united surgeons from 116 counties and rapidly produced patient-level data which changed practice across the world. The first protocol was developed in 3 days, and the results were published in 30 days from completion, influencing every type of surgeon. In addition, the collaborative model means that the engagement of international leaders and stakeholders was incorporated into study development from the beginning, which translated into widespread adoption and ease of dissemination at the end. Closing the gap between publication and global practice change is a central function of this type of research.

Inclusivity and engagement of local collaborators is another key strength to collaborative research. This brings numerous benefits, including incorporating a variety of points of view, encouraging ownership, and expanding and diversifying expertise. Most importantly, engagement shifts the balance of power from external organizations and agencies back into the hands of local communities, patient groups, and collaborators. Listening to the collaborators in this two-way dynamic interaction is key to understanding the most pressing research needs. This provides a voice for them to influence international studies on behalf of their patients, which can be particularly important in areas of the world where researchers are under-represented and have limited access to social, political, and financial resources.

The authorship model that supports collaborative research is a secondary benefit to the patient gains, but does represent an impartial approach to handling individual contributions. The model supports a system whereby only the study group name appears on the top line of publications and all collaborators are listed together at the end. This model abandons the notion of attributing merit to a small group of individuals and leans towards inclusivity of everyone who worked towards the study. Fundamentally, this levels the playing field between the highest-ranking academics and most junior researchers, and encourages social mobility within elitist academic circles. An example of the effect of this authorship model was when the COVIDSurg group was recently awarded the Guinness World Record for the most collaborators on a scientific paper (of any type), with 15 000 authors. Of course, some of these collaborators did more than others, but that is also true of single-centre papers with 10 top-line authors, which deliver none of the wide-scale benefits to patients.

The legacy of collaborative work is that the networks can be transformed to become sustainable, fundable, and evergreen. The connections and infrastructure can be set up to remain intact after the primary study has finished and can address new research topics.

The single-centre, retrospective study of extravagant, highly subspecialized surgical techniques based on small case series is dead. We must recognize that the time and energy spent by researchers and staff is better served in answering bigger questions that will translate into tangible improvements in patient care on a national or global scale. As a community, we need to put patient benefit first, and fully support multicentre, prospective, collaborative research.

*Disclosure*. The authors declare no conflict of interest.
